# Green synthesis of metallic nanoparticles using pectin as a reducing agent: a systematic review of the biological activities

**DOI:** 10.1080/13880209.2021.1910716

**Published:** 2021-04-27

**Authors:** Kogilavanee Devasvaran, Vuanghao Lim

**Affiliations:** Integrative Medicine Cluster, Advanced Medical and Dental Institute, Universiti Sains Malaysia, Bertam, Malaysia

**Keywords:** Polysaccharide, rhamnogalacturonan I, antibacterial, anticancer, drug carrier, antioxidant, wound healing, anti-inflammatory

## Abstract

**Context:**

Pectin is a plant heteropolysaccharide that is biocompatible and biodegradable, enabling it to be an excellent reducing agent (green synthesis) for metallic nanoparticles (MNPs). Nevertheless, in the biological industry, pectin has been left behind in synthesising MNPs, for no known reason.

**Objective:**

To systematically review the biological activities of pectin synthesised MNPs (Pe-MNPs).

**Methods:**

The databases Springer Link, Scopus, ScienceDirect, Google Scholar, PubMed, Mendeley, and ResearchGate were systematically searched from the date of their inception until 10^th^ February 2020. Pectin, green synthesis, metallic nanoparticles, reducing agent and biological activities were among the key terms searched. The data extraction was focussed on the biological activities of Pe-MNPs and reported following the Preferred Reporting Items for Systematic Reviews and Meta-Analyses (PRISMA) recommendations for systematic reviews.

**Results:**

A total of 15 studies outlined 7 biological activities of Pe-MNPs in the only three metals that have been explored, namely silver (Ag), gold (Au) and cerium oxide (CeO_2_). The activities reported from the *in vitro* and *in vivo* studies were antimicrobial (9 studies), anticancer (2 studies), drug carrier (3 studies), non-toxic (4 studies), antioxidant (2 studies), wound healing (1 study) and anti-inflammation (1 study).

**Conclusions:**

This systematic review demonstrates the current state of the art of Pe-MNPs biological activities, suggesting that Ag and Au have potent antibacterial and anticancer/chemotherapeutic drug carrier activity, respectively. Further *in vitro*, *in vivo,* and clinical research is crucial for a better understanding of the pharmacological potential of pectin synthesised MNPs.

## Introduction

Green synthesis is described as the eco-friendly method of synthesising nanoparticles using plant, plant compounds, or microbial resources rather than harmful chemicals as a reducing agent (Park 2014). Pectin extracted from the middle lamella and cell walls of plants is soluble in water, making it a vital reducing agent for the synthesis of nanoparticles (Voragen et al. 2009; Daher & Braybrook 2015; Rana et al. 2019). Furthermore, due to its availability, cost-effectiveness, non-toxic, biocompatible, and biodegradable nature (Liu et al. 2003; Das et al. 2011; Meneguin et al. 2014; Devendiran et al. 2016; Kumari et al. 2016), pectin is often studied for various purposes.

**Figure 1.**
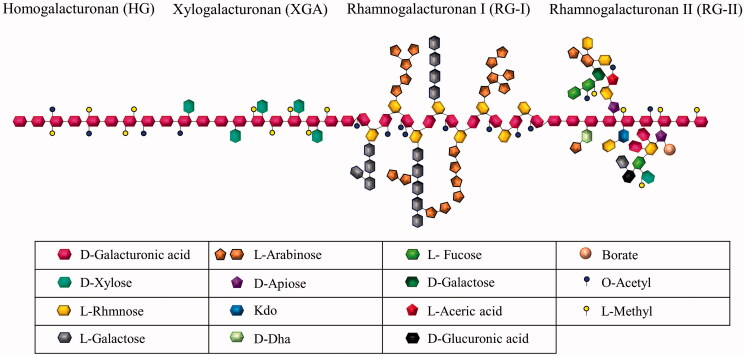
A schematic representation of pectin; homogalacturonan (HG), xylogalacturonan (XGA), rhamnogalacturonan I (RG-I) and rhamnogalacturonan II (RG-II) regions.

The pectin schematic structure (Figure 1) consists of a homogalacturonan (HG) backbone, xylogalacturonan (XGA), rhamnogalacturonan I (RG-I) and rhamnogalacturonan II (RG-II) regions. The pectin foundation comprises of acetylated and methylated α (1–4)-galacturonic acid units. The HG region is the most abundant and stretches up to 100 GalA, comprising approximately 60% of the pectin. The XGA region differs from HG only by substituting O-3 with β-linked xylose (Mohnen 2008).

**Figure 2.**
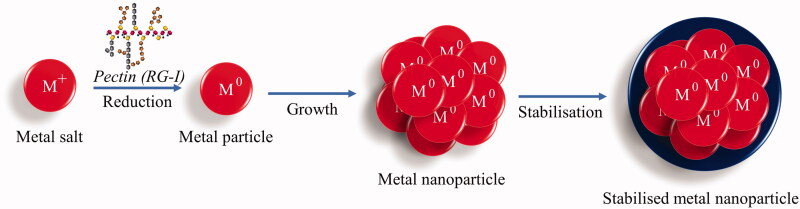
Mechanism of pectin synthesising metallic nanoparticles.

The RG-I region makes up approximately 20–35% of pectin and is composed of arabinan and galactan side chains, which contain hydroxyl groups (Mohnen 2008; Hileuskaya et al. 2020). Due to the shift of the tautomeric equilibrium (cyclo-oxo-tautomerism), the free hemiacetal hydroxyl groups may be converted to free aldehyde groups in an alkaline medium. The reducing properties of pectin macromolecules are provided by these aldehyde groups (Hileuskaya et al. 2020). Thus, RG-1 reduces metal salts to metal nanoparticles (Figure 2), enabling pectin to reduce metallic nanoparticles (MNPs) and form pectin metallic nanoparticles (Pe-MNPs). The RG-II region, however, is the most complex and is made up of some of the rarest moieties, such as 3-deoxy-d-lyxo-2-heptulosaric acid (DHA), 3-deoxy-d-manno-2-octulosonic acid (Kdo), aceric acid, fucose, and apiose (Tan et al. 2018). This region has contributed to several studies, including mitogenic activity and immune complexes clearance enhancing activity (Shin et al. 1997; Sakurai et al. 1999).

MNPs are inorganic nanoparticles within the range of 1–100 nm made of pure metals or their compounds (Bhattacharya & Mukherjee 2008; Venkatesh et al. 2018; Pinon-Segundo et al. 2019). To date, several MNPs, including cerium oxide (CeO_2_) (Patil et al. 2016), copper (Cu) (Venkatakrishnan et al. 2014), gold (Au) (Nigoghossian et al. 2015; Ahmed et al. 2016), iron (Fe) (Ngenefeme et al. 2013), palladium (Pd) (Khazaei et al. 2013; Baran 2018), platinum (Pt) (Hikosaka et al. 2008), selenium (Se) (Pornwilard et al. 2014), silver (Ag) (Kong et al. 2008; Al-Muhanna et al. 2015; Nigoghossian et al. 2015), titanium oxide (TiO_2_) (Dash et al. 2019) and zinc (Zn) (Pistone et al. 2017) have been synthesised using pectin as reducing agent.

Although all these metals have used pectin as their reducing agent, the biological activities of only three metals, namely Ag, Au, and CeO_2_, have been reported. This systematic review focuses on the green synthesis of MNPs using pectin as a reducing agent and the biological activities of Pe-MNPs in its antimicrobial, anticancer, drug carrier, non-toxic, antioxidant, wound healing, and anti-inflammatory activities.

## Methods

**Figure 3.**
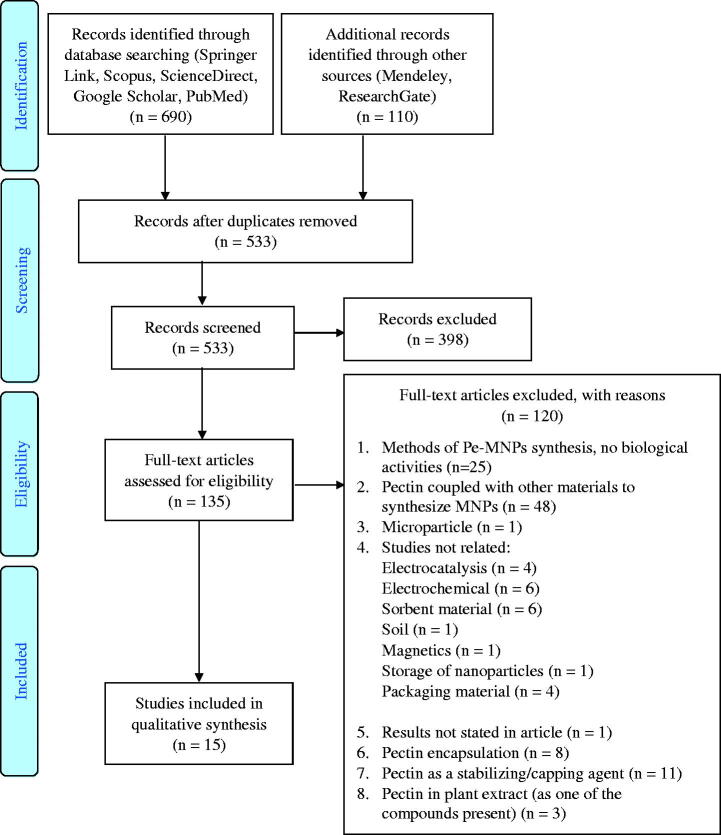
Flow diagram of study selection adapted from the PRISMA group (Moher et al. 2009).

In this systematic review, all published data were searched and collected from inception until 10 February 2020, using multiple electronic databases according to the Preferred Reporting Items for Systematic Reviews and Meta-Analyses (PRISMA) recommendations for systematic reviews (Moher et al. 2009). The electronic search was conducted on Springer Link, Scopus, ScienceDirect, Google Scholar, Mendeley, PubMed and ResearchGate using the following keywords: Green synthesis, Pectin, Metallic, Silver, Gold, Copper, Platinum, Titanium, Selenium, Cerium, Palladium, Boron, Iron, Zinc, Capping, Stabilising, Reducing, Nucleation and Nanoparticles. Additional keywords were chosen for biological activities: antioxidant, anti-inflammatory, anticancer, drug delivery, and wound healing. The following inclusion criteria were used to obtain a more specific search result: pectin as a reducing agent (green synthesis), biological activities, articles accepted or published with availability in electronic databases by 10 February 2020 and articles only in English. The articles that focussed on encapsulation/entrapment of pectin, extract of plant/fruit containing pectin as one of the compounds, pectin as a stabilising and/or capping agent only and studies not related to green synthesis or biological activities were excluded. The information and data extraction were focussed on the green synthesis of Pe-MNPs and biological activities. The specific applications featured reduction of pectin synthesised MNPs, such as the particle size and types of biological activities. After the search, two independent examiners screened and reviewed the research titles and abstracts. The data collection, management, and analysis of all relevant evidence for Pe-MNPs is presented in the flow diagram (Figure 3).

The synthesis method keywords were not included during the search since articles about Pe-MNPs with biological activities only were included in this review, excluding all other articles on Pe-MNPs with other uses such as electrochemical sensors, electrocatalysis, sorbents, soil management, magnetics, and food packaging.

## Results

Following the search of electronic databases, the yields were as follows: Springer Link − 273 articles, Scopus − 197 articles, ScienceDirect − 93 articles, Google Scholar − 88 articles, Mendeley − 87 articles, PubMed − 39 articles, and ResearchGate − 23 articles. All the articles were imported into Mendeley, and the results from the databases were merged, obtaining 800 articles. Using a duplicate removal tool (Mendeley), 267 duplicates were removed. Screening of the abstracts of the 533 remaining articles identified 398 unrelated articles. Of the remaining 135 articles, 15 studies met the inclusion criteria, and 120 studies were excluded.

Therefore, 15 studies were included in the qualitative synthesis. Eight studies focussed on pectin synthesised silver nanoparticles (Pe-AgNPs), six studies on pectin synthesised gold nanoparticles (Pe-AuNPs), and one study focussed on pectin synthesised cerium oxide nanoparticles (Pe-CeO_2_NPs) that reported biological activities. Manuscripts that evaluated more than one biological activity were divided and assigned to the relevant designated category.

### Antimicrobial activities

Antimicrobial activities of Pe-MNPs are the most researched biological activity to date (Table 1). Ag is the most common metal used among Pe-MNPs to study antibacterial activity, with only one activity reported from another metal (CeO_2_). The first study in this field, which was conducted by Balachandran in 2013, reported that the supplementation of Pe-AgNPs had damaged the cellular membrane of *Escherichia coli* cells with a minimum inhibitory concentration (MIC) of 30–40 µg in nutrient agar and 60 µg in nutrient broth, respectively.

**Table 1.** Antimicrobial activities of pectin synthesised metallic nanoparticles.

Metal/Metal oxide	Pectin source	Degree of esterification (^a^LM or ^b^HM)	Role of pectin in NP synthesis	Diameter (nm)	Shape	Microbial strain	^c^MIC/^d^MBC/% Survival/Zone of Inhibition	Reference
Cerium oxide	Indian red pomelo fruit peel	79.04% (HM)	Reducing and stabilising agent	2–40	Spherical	*E. coli*, *B. subtilis*	% Survival at different concentrations; At 1 mM *E. coli − 30*% *B. subtilis*- 40% At 2 mM *E. coli* − 5% *B. subtilis* − 10%	Patil et al. 2016
Silver	Apple peel (Sigma Aldrich)	^e^NA	Reducing and capping agent	9.3	Spherical	*E. coli*	MIC In nutrient agar; 30-40 µg In nutrient broth; 60 µg	Balachandran et al. 2013
Silver	Commercial (Merck, India)	^e^NA	Reducing agent	∼20–40	Ring shape	*E. coli*, *B. subtilis*	Zone of inhibition; *E. coli*- 10.3 ± 0.7 mm *B. subtilis − 15.3* ± 0.5 mm	Rao et al. 2015
Silver	Citrus peel (Sigma Aldrich)	6–7 % (LM)	Reducing agent	∼8	Spherical	*E. coli, S. epidermidis*	MIC At 6 h; *E. coli* − 15.62 µg *S. epidermidis* − 250 µg At 24 h; *E. coli* − 31.25 µg *S. epidermidis* − 500 µg	Pallavicini et al. 2017
Silver	Citrus peel (Sigma Aldrich)	^e^NA	Reducing and capping agent	3	Spherical	*E. coli*, *S. aureus*	MIC *E. coli* & *S. aureus* − 6.25-12.15 mg/L	Zhang et al. 2017
Silver	Commercial (Sigma Aldrich)	80.4 % (HM) & 37.5% (LM)	Reducing and capping agent	8–28	Spherical	*E. coli*, *B. pumilus*, *B. subtilis*	MIC (HM & LM) *E. coli − 0.18*–0.39 mM *B. pumilus* & *B. subtilis − 0.39*–1.55 mM	Hileuskaya et al. 2020
Silver	Apple peel (Sigma Aldrich)	70.2 % (HM)	Reducing agent	450	Nanofiber	*E. coli*	^e^NA	Li et al. 2018
Silver	Orange peel	^e^NA	Reducing agent	2.90 & 11.94	Spherical	*E. coli*, *S. aureus*, *Aspergillus japonicus*	MIC *E. coli* & *S. aureus − 80*–160 mg/mL Zone of inhibition (At different NP sizes); At 2.9 nm (Pe-AgNPs); *E. coli* − 9.8 mm *S. aureus* − 10 mm *A. japonicus − 19.4* mm At 11.94 nm (Pe-AgNPs); *E. coli* − 10 mm *S. aureus* − 11.6 mm *A. japonicus − 19.3* mm	Su et al. 2019

^a^LM refers to Low Methoxyl Pectin, ^b^HM refers to High Methoxyl Pectin, ^c^MIC: Minimum Inhibitory Concentration, ^d^MBC: Minimum Bactericidal Concentration and ^e^NA refers to Not Available.

In 2015, Rao et al. studied the activity of Pe-AgNPs on Gram-positive bacteria (*Bacillus subtilis)* in contrast to Gram-negative bacteria (*E. coli*). Their results confirmed that the Gram-positive inhibition zone (15.3 ± 0.5 mm) was larger than Gram-negative (10.3 ± 0.7 mm) bacteria and concluded that Pe-AgNPs film showed effective antimicrobial activity on both strains. Su et al. (2019) reported the MIC of Gram-positive and Gram-negative bacteria to be in a range of 80–160 mg/mL, with a slightly larger inhibition zone (10 and 11.6 mm) in both sizes (2.9 and 11.94 nm) of Pe-AgNPs tested in the Gram-positive bacteria. However, several studies reported that Pe-AgNPs had a lower MIC in Gram-negative compared to Gram-positive bacteria (Patil et al. 2016; Pallavicini et al. 2017; Zhang et al. 2017; Hileuskaya et al. 2020).

**Figure 4.**
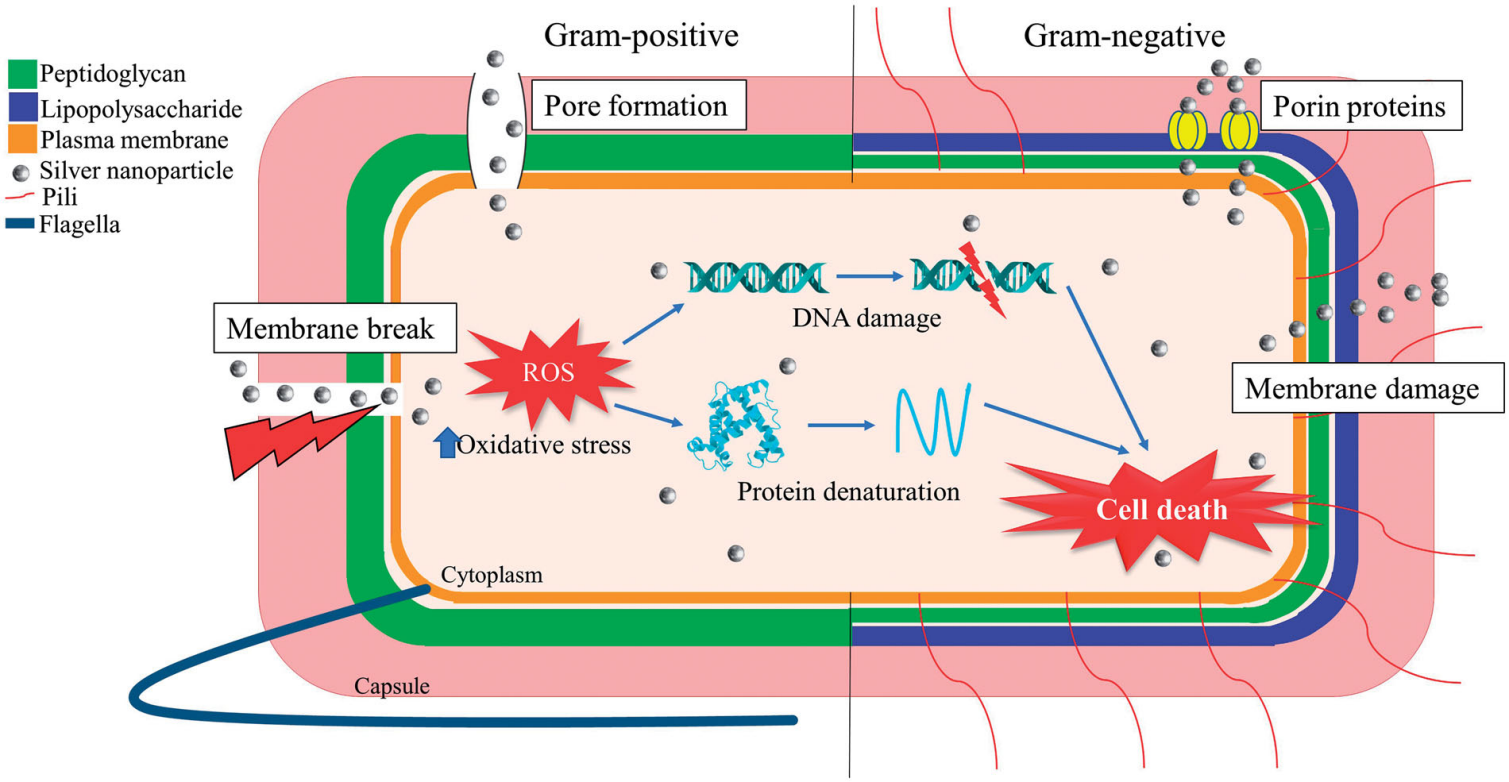
Possible mechanism of action of pectin synthesised silver nanoparticles on Gram-positive and Gram-negative bacteria. *ROS represents reactive oxygen species.

Hileuskaya et al. (2020) reported an interesting discovery, where low methoxy (LM) and high methoxy (HM) pectin synthesised AgNPs had a different activity. Among the 3 strains of bacteria tested, HM_Pe-AgNPs showed elevated activity against *B. pumilis*, *B. subtilis*, and *E. coli*, while LM_PeAgNPs only had an elevated activity against *B. subtilis*. However, this study did not state an exact reason and concluded significant Pe-AgNPs activity in Gram-negative bacteria (*E. coli)* with a MIC of 0.18–0.39 mM compared to Gram-positive bacteria (*Bacillus* sp.) with a MIC of 0.39–1.55 mM. The difference in activity between Pe-AgNPs in Gram-positive and Gram-negative bacteria is due to the structural difference in their cell wall (Figure 4).

The cell wall of the Gram-negative bacteria consists of an outer membrane with lipopolysaccharide (LPS) and a thin peptidoglycan (PG) layer, whereas the Gram-positive bacteria have a very thick PG layer (Slavin et al. 2017). In the Gram-negative bacteria, AgNPs are absorbed by the LPS and cause direct damage to the PG layer, leading to increased membrane permeability, thus killing the bacteria via the diffusion of released Ag ions into the cytosol. However, in Gram-positive bacteria, the AgNPs directly penetrate through the thick PG layer to cause an Ag ion leakage into the cytosol (Xu at al. 2019), enhancing the bactericidal activity.

Li et al. (2018) reported the antibacterial activity for Ag nanofibers synthesised using pectin. The nanofiber size was 450 nm and used for the sustained release of the drug. The composite nanofibers could inhibit *E. coli* for 7 days and release Ag for 4 weeks, showing tremendous potential as a long-term antibacterial drug.

Following these substantial antibacterial results, another microbial target reported is antifungal activity. In 2019, Su et al. demonstrated the inhibitory zones of Pe-AgNPs (19.3 and 19.4 mm) on an *Aspergillus japonicus* strain, suggesting that the potent antifungal activity could be due to the inhibition of conidial germination. However, the molecular mechanism of the antifungal activity was not reported.

### Anticancer effects

**Figure 5.**
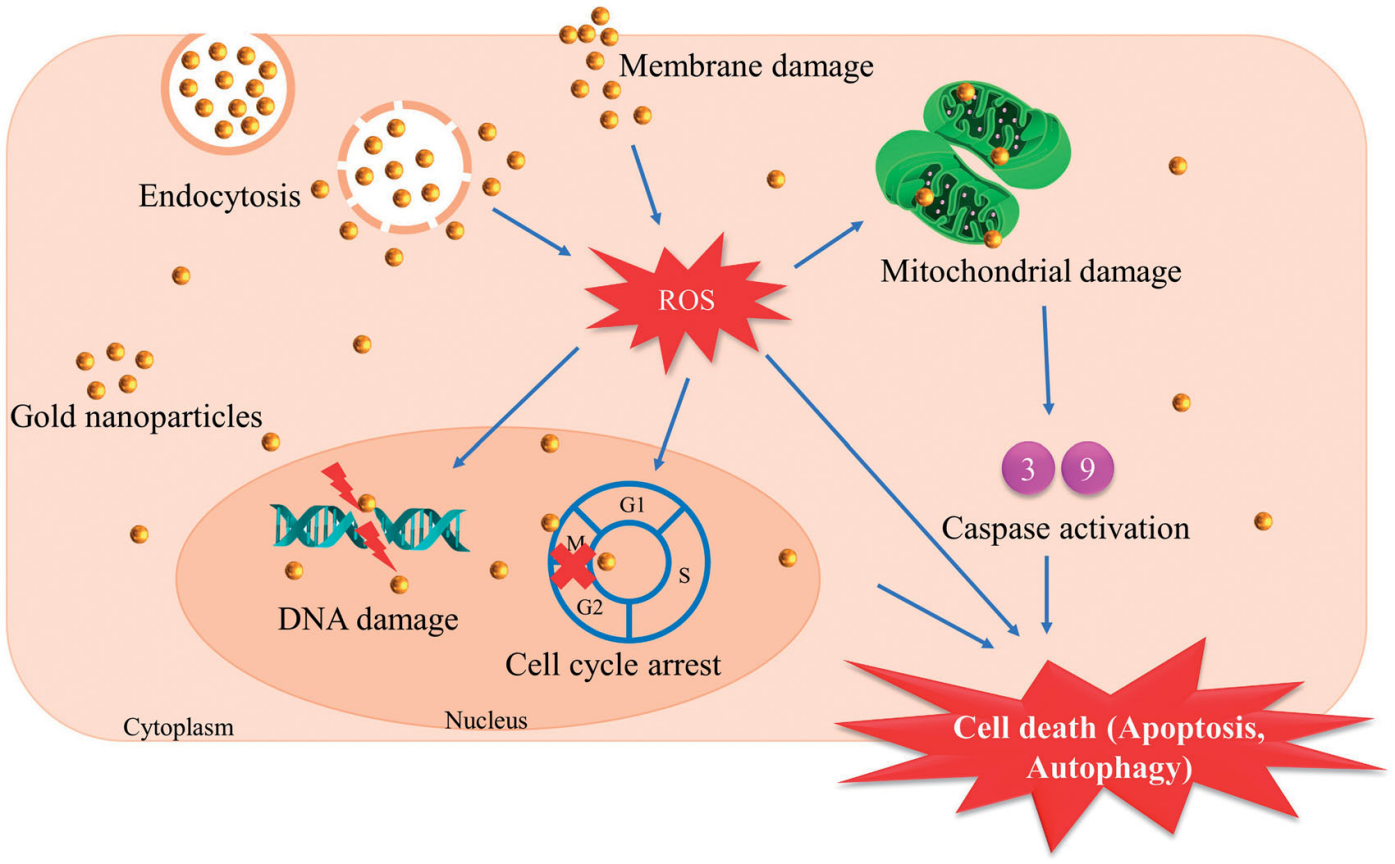
Possible mechanism of action of pectin synthesised gold nanoparticles on cancer cells. *ROS represents reactive oxygen species.

Several studies reported Pe-MNPs anticancer effects via the unloading of Au (Figure 5) or Ag from the Pe-MNPs (Table 2). Suganya et al. (2016) reported that Pe-AuNPs induced DNA damage in two breast cancer cell lines (MCF-7 and MDA-MB-231) via the comet assay. The DNA lesions drastically increased the comet tails length at the IC_50_ concentration (MCF-7 at 8 µg/mL and MDA-MB-231 at 2 µg/mL), suggesting cell death occurred from the fragmentation of DNA.

**Table 2.** Anticancer activities of pectin synthesised metallic nanoparticles.

Metal	Pectin source	Degree of esterification (^a^LM or ^b^HM)	Role of pectin in NP synthesis	Diameter (nm)	Shape	*In vitro* model (cells)	Function	IC_50_ Concentration	Reference
Gold	Classic cu701 (Herbstreith & Fox KG)	(LM)	Reducing, stabilising and capping agent	14	Spherical	HepG2, Hela	Drug carrier	In *HepG2;* ^c^DOX alone- 4.11 mg/mL Pe-AuNPs + DOX − 0.74 mg/mL In *Hela;* DOX alone- 3.88 mg/mL Pe-AuNPs + DOX − 3.27 mg/mL	Borker & Pokharkar 2018
Gold	Commercial (Sigma Aldrich)	^e^NA	Reducing, stabilising and capping agent	∼34	Spherical	HT-29	Drug carrier	DOX alone − 483 nM Pe-AuNPs + DOX − 351 nM ^d^FA-Pe-AuNPs + DOX − 240 nM	Devendiran et al. 2016
Gold	*Musa paradisiaca* (Banana)	(HM)	Reducing and stabilising agent	8	Spherical	MCF-7, MDA-MB-231	Drug	*MCF-7 − 8* µg/mL *MDA-MB-231 − 2* µg/mL	Suganya et al. 2016
Silver	Citrus peel (Sigma Aldrich)	^e^NA	Reducing, stabilising and capping agent	26	Spherical	EAC, Caco	Drug	*EAC − 35* µg/mL *ACO* − 39.5 µg/mL	Ghorab et al. 2016

^a^LM refers to Low Methoxyl Pectin, ^b^HM refers to High Methoxyl Pectin, ^c^DOX refers to Doxorubicin, ^d^FA refers to Folic acid and ^e^NA refers to Not Available.

Furthermore, the exposure of phosphatidylserine (PS) residues on the outer membrane were measured as an indicator of apoptosis. Suganya et al. (2016) employed a staining method, Annexin V-FITC, to stain PS, indicating apoptosis and propidium iodide (PI), a nuclear stain indicating necrosis. Pe-AuNPs stained double-positive green with Annexin V-FITC and red with PI, indicating apoptosis was induced in viable MCF-7 and MDA-MB-231 cells. This study concluded that Pe-AuNPs mediated apoptosis induction of MCF-7 and MDA-MB-231 cells were via increasing the sub-G1 population, leading to the DNA damage of cells.

Ghorab et al. (2016) studied the activity of natural polymers, namely pectin, chitosan, and alginate, with different γ irradiation doses to synthesise AgNPs and tested it on Ehrlich ascites carcinoma (EAC) cells and Human colon adenocarcinoma (Caco) cells. Pe-AgNPs reported the best growth of NPs with the highest stability among the three polymers tested. Pe-AgNPs at 5 kGy were biocompatible and induced a concentration-dependent inhibition of EAC and Caco cells. Ghorab et al. (2016) suggested that Pe-AgNPs may have induced changes in cellular functions, which led to a high hydrophobicity in the bovine haemoglobin that caused a transition of alpha helixes to beta sheets and led to the partial unfolding and aggregation of the protein. This study concluded that Pe-AgNPs exhibited anticancer activities.

### Drug carrier systems

Recently Pe-AuNPs have gained attention as a drug carrier system to promote the delivery of doxorubicin (DOX) (chemotherapeutic drug) and zidovudine (AZT) (antiretroviral drug). In 2016, Devendiran et al. reported Pe-AuNPs loaded with DOX enhanced the *in vitro* cytotoxicity of colon cancer (HT-29). The cationic DOX loaded on the anionic Pe-AuNPs exhibited excellent stability (-21.3 mV) at different pH levels and electrolytic conditions with a pH-dependent sustained release of DOX. Folic acid (FA) was conjugated to the DOX-loaded Pe-AuNPs to provide a cell-specific delivery as HT-29 cells are folate receptor-positive. This study reported that the proportion of cells from 36.21% in Pe-AuNPs increased to 78.24% in the G2/M phase after treatment with FA_Pe-AuNPs + DOX, indicating G2/M phase arrest, making it a promising drug carrier targeting colon cancer.

Borker and Pokharkar (2018) reported that Pe-AuNPs loaded with DOX exhibited excellent stability (-21.64 mV) under varying pH and electrolyte conditions, similar to the study mentioned above (Devendiran et al. 2016). However, this study reported the effects of Pe-AuNPs loaded with DOX on human liver cancer cells (HepG2 cells*),* overexpressing the asialoglycoprotein receptor (ASGPR). A lack of cytotoxicity was observed in HepG2 cells and HeLa cells, where > 85% of viable cells were reported after 48 h of receiving Pe-AuNPs. The non-cytotoxic trait of Pe-AuNPs is attributed to the biocompatible nature of pectin, the stability of Pe-AuNPs in the intracellular environment and a lack of anionic AuNP interaction with the negatively charged cell membrane (Goodman et al. 2004).

Pe-AuNPs loaded with DOX showed concentration-dependent cytotoxicity. The viability of HepG2 and HeLa cells decreased with increased concentration of DOX (0.01–5 µg/mL). However, the results showed a significant difference between the DOX and Pe-AuNPs + DOX in HepG2 cells and not in HeLa cells because the ASGPR receptor is expressed in HepG2 cells. This study suggested that Pe-AuNPs could be a vital anticancer drug carrier to treat hepatocellular carcinoma.

Another activity reported is targeted antiretroviral drug carrier of Pe-AuNPs via the encapsulation of AZT. The Pe-AuNPs were taken up by the macrophages (RAW 264.7 cells), suggesting that this method could reduce the toxicity of AZT being exposed to normal tissues (Borker et al. 2017). Interestingly, there was a slight increase in the survival rate (∼20%) of cells receiving AZT-Pe-AuNPs compared to AZT solution at a concentration of 1.25 mM. This result demonstrated that the cytotoxicity of the drug-loaded in Au nanoparticles was reduced due to the non-cytotoxic nature of Pe-AuNPs, which could be attributed to the points mentioned above (Goodman et al. 2004).

To better evaluate the *in vitro* results, an *in vivo* study using male Wistar rats was performed. The results for Pe-AuNPs and AZT-Pe-AuNPs were similar after 24 h, with the highest amount of Au present in the liver (∼70%), followed by spleen (∼3%), lymph nodes (∼3%), blood (∼0.5%), lungs (∼0.5%) and kidney (∼0.4%). The preferential localisation of Pe-AuNPs was the liver due to the presence of galactose residues in pectin. The galactose residues bound specifically to the ASGPR receptor on the surface of hepatocytes and led to an increased uptake via the receptor-mediated endocytosis (Yik et al. 2002). The increased AuNP uptake in the lymph nodes and spleen was due to the preferred uptake of Pe-AuNPs by the MGL1 receptor expressed in macrophages and dendritic cells (Kawasaki et al. 1986). This observation, coupled with the *in vitro* cellular uptake results, led to the conclusion that the Pe-AuNPs were taken up by macrophages. Thus, Pe-AuNPs can prove useful for targeting viral reservoir sites.

### Toxicity studies

Patil et al. (2016) studied the cytotoxicity of Pe-CeO_2_NPs via an erythrocyte haemolysis assay (Table 3). The results suggested that Pe-CeO_2_NPs are biocompatible in nature. However, the haemolysis increased (0.55–8.31%) with increasing concentrations (0.05–8.00 mg/mL) of Pe-CeO_2_NPs. Since the permissible limit of haemolysis set for biocompatibility assessments of material/biomaterial is 5% (Singhal and Ray 2002), this study reported a ≤ 4 mg/mL concentration of Pe-CeO_2_NPs (4.55% haemolysis), exhibited minimal cytotoxicity and is considered safe for human beings.

**Table 3.** Other biological properties of pectin synthesised metallic nanoparticles.

Metal/ Metal oxide	Pectin source	Degree of esterification (^a^LM or ^b^HM)	Role of pectin in NP synthesis	Diameter (nm)	Shape	Biological activity	Sample	Reference
Cerium oxide	Indian red pomelo fruit peels	79.04% (HM)	Reducing and stabilising agent	2–40	Spherical	Antioxidant, non-cytotoxic	Erythrocyte	Patil et al. 2016
Silver	Citrus peel (Sigma Aldrich)	^c^NA	Reducing, stabilising and capping agent	26	Spherical	Antioxidant	EAC cells	Ghorab et al. 2016
Gold	*Musa paradisiaca* (Banana)	(HM)	Reducing and stabilising agent	8	Spherical	Toxicity study	Sprague– Dawley rats	Suganya et al. 2017
Gold	Commercial (Sigma Aldrich)	^c^NA	Reducing, stabilising and capping agent	∼34	Spherical	Toxicity study	Zebrafish embryo	Devendiran et al. 2016
Gold	Orange peel	^c^NA	Reducing and stabilising agent	7–13	Spherical	Anti-inflammatory	Vero cells	Reena et al. 2017
Gold	Classic cu701 (Herbstreith & Fox KG)	32–38% (LM)	Reducing and stabilising agent	13	Spherical	Antiretroviral drug carrier	RAW 264.7 cells**Male Wistar rats	Borker et al. 2017
Silver	Citrus peel (Sigma Aldrich)	6–7% (LM)	Reducing agent	∼8	Spherical	Non-cytotoxic, wound healing	NHDF cells	Pallavicini et al. 2017

^a^LM refers to Low Methoxyl Pectin, ^b^HM refers to High Methoxyl Pectin and ^c^NA refers to Not Available.

Devendiran et al. (2016) measured the toxicity of Pe-AuNPs through a Zebrafish toxicity study. Zebrafish embryos were employed to study the toxicity effects Pe-AuNPs, to which no malformations in the embryos were observed, concluding an absence of toxic effects upon hatching of the Zebrafish. A 100% survival rate of Zebrafish was reported at all Pe-AuNPs (200–1000 ng/mL) concentrations tested. This study suggested that Pe-AuNPs are highly suitable for biomedical and drug delivery applications.

In 2017, Suganya et al. conducted a study that explored the acute and sub-acute toxicity of Pe-AuNPs in Sprague-Dawley rats. The acute toxicity study reported no mortality, organ damage or abnormalities in the animal necropsies, concluding that Pe-AuNPs would be orally safe at a single dosage of 5 and 10 mg/kg. The subacute toxicity results indicated no abnormal changes or significant adverse effects on the animal after a continuous dose administration for 4 weeks. The *in vivo* acute and sub-acute toxicity studies suggested that Pe-AuNPs are safe at the sub-acute level with no significant toxicity (Suganya et al. 2017).

Pallavicini et al. (2017) studied the cytotoxic activity of Pe-AgNPs (0.001 M Ag in 1.0% pectin) in fibroblast cells (NHDF cells). This study compared the cytotoxicity of NHDF cells between Pe-AgNPs and pure pectin (1% aqueous pectin) against the medium that is not supplemented with foetal bovine serum. The results reported at a dilution of 1:20, Pe-AgNPs had a viability percentage of 120–140%, while pure pectin had a viability percentage of 105–110%, which was comparable to the medium supplemented with bovine serum. The results concluded that Pe-AgNPs are not cytotoxic and enhances the viability of NHDF cells.

### Antioxidant effects

Antioxidant activities of Pe-CeO_2_NPs and Pe-AgNPs using 2,2-diphenyl-1-picrylhydrazyl (DPPH) were reported in two separate studies (Ghorab et al. 2016; Patil et al. 2016). The DPPH radical scavenging capacity of Pe-CeO_2_NPs (4.0 mg/mL) was up to 73% in 60 min. The antioxidant activity increased with the increase in Pe-CeO_2_NPs concentration. The IC_50_ value was reported at a concentration of 1.83 mg/mL. This study suggested that CeO_2_ possesses a fluorite crystalline structure responsible for the redox reaction on the surface of the NPs, promoting antioxidant activity (Korsvik et al. 2007). The Pe-CeO_2_NPs can regenerate antioxidant activity via having both catalase and superoxide dismutase mimetic activity (Soren et al. 2015).

The Pe-AgNPs in Ghorab et al. (2016) study used γ irradiation at different doses to synthesise the nanoparticles. The findings suggested that the antioxidant activity of Pe-AgNPs decreased with an increase in γ irradiation. At a radiation dose of 5 kGy, which was the dose used in this study, the Pe-AgNPs DPPH radical scavenging capacity was 60.67%, compared to the citrus pectin alone, which was 50.61%. The IC_50_ of Pe-AgNPs was reported at 10 kGy γ irradiation. Thus, this study indicated that the marginal increase in antioxidant activity of the synthesised Pe-AgNPs was due to the stabiliser and reducer itself, which is pectin, and not the AgNPs.

### Wound healing effect

Since Pe-AgNPs were demonstrated to be non-cytotoxic (120–140% viability) on NHDF cells, Pallavicini et al. (2017) went on to test the proliferative and wound healing properties via scratch-wound assay. The results were impressive because the proliferation rate of NHDF cells was 2-fold higher in complete medium (medium with serum) at 24 and 48 h compared to the Pe-AgNPs group, but at 72 h, both groups managed to close the gap in the scratch-wound assay. The findings could suggest that Pe-AgNPs exhibited a time response activity. Pe-AgNPs promoted cytokine regulation, which alleviated the healing of fibroblast colonies. This study suggested that the weakly interacting oxygen molecules with the Ag surface increased the NHDF cells viability. Therefore, concluding that Pe-AgNPs can be used as a pre-treatment to prevent bacterial activity and promote implant surgery recovery.

### Anti-inflammatory effect

Pectin and AuNPs exhibited anti-inflammatory effects in previous studies (Ovodova et al. 2009; Popov et al. 2005; Ghanizadeh 2012). The anti-inflammatory activity of pectin is reported to be mainly contributed by the galacturonan backbone (Markov et al. 2011), while the AuNPs is via the inhibition of inflammatory cytokines (Chen et al. 2013). Reena et al. (2017) studied the anti-inflammatory effects of Pe-AuNPs in contrast to Pe-AuNPs-PLA-PEG-PLA nanoconjugates via membrane stabilisation and protein denaturation in African green monkey’s kidney cell line (Vero cells). The results at 200 µg/mL Pe-AuNPs reported a protein denaturation inhibitory activity of 58.2%, while the Pe-AuNPs-PLA-PEG-PLA reported inhibition of 63.1%. Similarly, the membrane stabilisation activity at 200 µg/mL reported being 60.1% in Pe-AuNPs and 64.1% in Pe-AuNPs-PLA-PEG-PLA. Although no significant difference between the two groups could be observed, the study suggested that the conjugation of PLA-PEG-PLA enhanced the anti-inflammatory activity of Pe-AuNPs.

## Discussion

This systematic review presents the key findings of the biological activities explored using pectin as a reducing agent for the synthesis of MNPs. It also provides an overview of the types of MNPs explored and the shapes and sizes used. Detailed documentation of information retrieved from articles enables other researchers to verify the validity of the findings.

MNPs have been explored for decades in the biological field; however, there is some evidence that MNPs contribute to liver toxicity (Yao et al. 2019) with certain contributing factors, namely MNPs size and the amount of metal stored in the liver upon excretion (*in vivo*). The issue of the toxicity of MNPs has been discussed but only to suggest that each metal has a different level of toxicity, where Ag and Au were shown to be safer than most other metals for biological studies (Bahadar et al. 2016).

Our findings identified that the majority of the biological studies employing Pe-MNPs used Ag and Au nanoparticles. A key factor is that the Food and Drug Administration or other regulatory bodies have approved Ag (Sood and Chopra 2018) and Au (Bobo et al. 2016) to be tested for use in biomedicine. Ag has been used diversely as an antibacterial agent, causing oxidative stress, DNA damage, protein denaturation and membrane damage (Brandelli et al. 2017). However, Au has been proven to have anticancer activities by inducing apoptosis, necrosis, and autophagy (Sun et al. 2018). These reasons support the choice of metals used by researchers to conduct studies using Pe-MNPs to assess the antibacterial and anticancer activities, which constituted most of the biological activities studied.

Although these metals have often been studied, the synthesis of MNPs with toxic or hazardous chemicals reduces the metal’s biocompatibility, effectivity, and safety in living beings (Das et al. 2017). Biosynthesis (living materials) is a method used to rapidly synthesise nanoparticles in an eco-friendly, non-toxic manner with the ability to control the size of the nanoparticle (Ghozali et al. 2015). Pectin is an ideal reducing agent soluble in water and abundant in many plant sources (Rana et al. 2019). Numerous bioactivities of this heteropolysaccharide have been reported, including anti-inflammatory, hypoglycaemic, immunoregulatory, antioxidant, antibacterial, and antitumor activities (Minzanova et al. 2018), which has led us to believe that the use of Pe-MNPs is advantageous in the biological field.

The shape of all the Pe-MNPs studied was spherical with a size of 40 nm and below except for one study reported on a nanotube with a size of 450 nm. The Pe-MNPs exhibited an excellent antibacterial and anticancer effect, which could be due to their small (<100 nm) size and large surface-to-volume ratio (Niazi and Gu 2009; Saeed et al. 2019).

The number of studies is not enough to obtain a definite idea of the biological activities of Pe-MNPs and the mechanism of actions. However, based on the results, it is evident that Pe-MNPs exhibit antimicrobial, anticancer, drug carrier, antioxidant, anti-inflammatory, wound healing, and non-cytotoxic properties, which may be dependent on different factors such as metal type, shape, and size. Nevertheless, more research should be conducted on various biological activities to understand the pharmacological potential of Pe-MNPs better.

## Conclusions

Based on the results and discussion above, it can be concluded that all the research conducted on Pe-AgNPs for the antibacterial activities and Pe-AuNPs for the anticancer and drug carrier activities exhibited positive results. Our systematic review concludes that Pe-MNPs did show potent biological activities with biocompatible and non-toxic nature, suggesting that Ag and Au are suitable metals synthesised by pectin. However, due to the limitation of studies conducted over the years, the biological activities of Pe-MNPs require further research in the *in vitro*, *in vivo*, and clinical fields to confirm their efficacy.
